# On comparative analysis of a two dimensional star gold structure via regression models

**DOI:** 10.1038/s41598-024-66395-1

**Published:** 2024-07-08

**Authors:** Muhammad Farhan Hanif, Hasan Mahmood, Shahbaz Ahmad, Mohamed Abubakar Fiidow

**Affiliations:** 1 Abdus Salam School of Mathematical Sciences, Government College University, Lahore, Pakistan; 2 Department of Mathematics, Government College University, Lahore, Pakistan; 3 Department of Mathematical Sciences, Faculty of Science, Somali National University, Mogadishu Campus, Mogadishu, Somalia

**Keywords:** Topological indices (TI), Entropy measure, Regression model, Star gold structure (SGN), Applied mathematics, Physical chemistry

## Abstract

In this research, the star gold structure with beta graphene is thoroughly examined. We mainly focus on computing degree-based topological indices, which provide information about the network’s connectivity and complexity as well as structural features. In addition, we compute an entropy measure to represent the uncertainty, information richness, and degree of unpredictability in the network. Furthermore, this study explores the relationships between topological descriptors and entropy using regression models that are logarithmic, linear, and quadratic. By merging these regression models, we uncover hidden patterns and understand the underlying ideas governing the network’s behaviour. Our findings shed light on the connection between topological indices and entropy. This work improves our understanding of star gold structure dynamics and provides a visual framework for interpreting their behaviour.

## Introduction

The mathematical structure known as the graph is composed of nodes, also known as vertices, joined by edges. The study of these structures is known as graph theory^1^. These graphs allow for the effective simulation and examination of the connections and links in a variety of systems, such as social networks, transportation networks, and more. Graph theory is an important area of study in computer science and mathematics because of its diversity and adaptability^2^. Among the various domains in which it finds application are computer networking, biology, and optimization. The vertex set is denoted by ${\mathfrak {V}}$ and the edge set is denoted by *E* of a graph *G*. The degree of the vertex ${\mathfrak {v}}$ is the number of edges incident to the vertex ${\mathfrak {v}}$ and it is represented as $\mathfrak {B_{v}}$.

In the chemical graph theory, atoms are represented as vertices, and chemical bonds between them as edges^3^. Often referred to as molecular graphs, these illustrations offer a useful tool for understanding the properties, makeup, and behaviour of chemical substances. The mathematical closed-form formulations for 2D nanotubes were developed by Asadullah et al.^4^, They discussed how they computed the entropy and indices of the nanotubes. Arockiarajet al.^5^ examined the comparison of pharmacological agents for the treatment of corona using linear and cubic regression models. Naeem et al.^6^ analyzed the physicochemical properties of benzene derivatives by modeling with curvilinear regression on the reverse entropy indices. Ye et al.^7^ discussed the entropy measures of some Titania and Carbon Nanotubes.

Topological indices are quantifiable indicators of the connection inside molecular networks. These indices provide a single numerical number that represents the molecular structure’s size, shape, branching, and symmetry. Their ability to quantify molecular topology aids in the prediction of several chemical properties and behaviours, including biological activity, toxicity, and bioavailability^8^. Several significant indices for $\gamma$-graphyne and Zigzag graphene nanoribbon were calculated by Hakeem et al.^9^. Huang et al.^10^ talked about QSPR analysis and molecular modeling of medications for Lyme disease. By examining the relationships between molecular structure and activity using topological indices, scientists can gain more insight into the basic mechanisms governing chemical interactions and develop more effective techniques for constructing molecules with specific biological or physicochemical properties. The following are the *TI* of the graph *G*: The Randic index, which was first described by Randic^11^, is defined as follows in Eq. (1):

1 $$\begin{aligned} \chi (G)=\sum _{\mathfrak {uv}\in E(G)}\frac{1}{\sqrt{\mathfrak {B_{u}}\mathfrak {B_{v}}}} \end{aligned}$$

The first and second Zagreb indices were defined by Gutman et al.^12^ and are defined as follows in Eqs. (2) and (3):

2 $$\begin{aligned} {M_{1}}(G)=\sum _{\mathfrak {uv}\in E(G)}{(\mathfrak {B_{u}}+\mathfrak {B_{v}})} \end{aligned}$$

3 $$\begin{aligned} {M_{2}}(G)=\sum _{\mathfrak {uv}\in E(G)}{(\mathfrak {B_{u}}\mathfrak {B_{v}})} \end{aligned}$$

Zhong^13^ introduced the harmonic index *HM* in 2012. Its definition is given in Eq. (4) as follows:

4 $$\begin{aligned} HM(G)=\sum _{\mathfrak {uv}\in E(G)}{(\mathfrak {B_{u}}+\mathfrak {B_{v}})^{2}} \end{aligned}$$

The atom-bond connectivity (*ABC*) index was introduced by Estrada^14^ and is defined in Eq. (5) as follows:

5 $$\begin{aligned} ABC(G)=\sum _{\mathfrak {uv}\in E(G)}\sqrt{\frac{\mathfrak {B_{u}}+\mathfrak {B_{v}}-2}{\mathfrak {B_{u}}\mathfrak {B_{v}}}} \end{aligned}$$

The sum-connectivity index of a graph, which is defined in Eq. (6) as follows, is a modified version of the connectivity index that Bo Zhou and Nenad Trinajstić^15^ introduced.

6 $$\begin{aligned} SCI(G)=\sum _{\mathfrak {uv}\in E(G)}\frac{1}{\sqrt{\mathfrak {B_{u}}+\mathfrak {B_{v}}}} \end{aligned}$$

The definition of the geometric arithmetic index is given in Eq. (7) and was initially presented by Furtula et al.^16^.

7 $$\begin{aligned} GA(G)=\sum _{\mathfrak {uv}\in E(G)}\frac{2\sqrt{\mathfrak {B_{u}}\mathfrak {B_{v}}}}{\mathfrak {B_{u}}+\mathfrak {B_{v}}} \end{aligned}$$

In graph theory, entropy measures are a way to gauge how complex or unpredictable a graph’s structure is. Graph entropy is a well-known entropy measure in graph theory. It is inspired by Shannon entropy in information theory. Graph entropy^17^ quantifies the level of uncertainty surrounding the vertex and edge distribution of a graph. By computing the graph entropy, researchers can assess the level of disorder or unpredictability in the graph’s structure^18^. Higher entropy networks have a greater diversity of subgraph patterns, which makes them more complex. Conversely, a graph with a lower entropy suggests that the configurations of the edges and vertices are more regular or orderly.

Graph entropy is used in many fields, such as biological network analysis, social network modeling, and network analysis^19^. It offers a systematic way to evaluate and compare the structural characteristics of various graphs, making it easier to comprehend their resilience, dynamics, and structure. Ghani et al.^20^ characterized chemical network entropies. Shanmukha et al.^21^ computed the degree-based entropy descriptors of graphenylene using topological indices. Arockiara et al.^22^ discussed the comparative examination of indices and entropy for medicinal compounds in the treatment of blood cancer. Ahmed et al.^23^ analyzed through python-based algorithmic approach to optimize sulfonamide drugs via mathematical modeling.

We give an edge-weight function definition for graph index-entropies^24^. Let $G=({\mathfrak {V}}, E)$ be a simple, undirected, connected graph of order *m*. Let ${\mathfrak {f}}$ be an arbitrary information functional. The entropy *W* of *G* is therefore defined as follows:

8 $$\begin{aligned} W_{{\mathfrak {f}}}(G)=-\sum _{i=1}^{m}\frac{\mathfrak {f(v_{i})}}{\sum _{i=1}^{m}\mathfrak {f(v_{i})}}\log \left( \frac{\mathfrak {f(v_{i})}}{\sum _{i=1}^{m}\mathfrak {f(v_{i})}}\right) \end{aligned}$$

After simplification of Eq. (8) we obtain the following expression of entropy: $W_{{\mathfrak {f}}}(G)$.

9 $$\begin{aligned} W_{{\mathfrak {f}}}(G)=\log (TI)-\frac{1}{(TI)}\sum _{\mathfrak {uv}\in E(G)}\mathfrak {f(uv)}\log {\mathfrak {f(\mathfrak {uv})}} \end{aligned}$$

Here *TI* means topological index, $\mathfrak {f(\mathfrak {uv})}$ means $\frac{1}{\sqrt{\mathfrak {B_{u}}\mathfrak {B_{v}}}}, {(\mathfrak {B_{u}}+\mathfrak {B_{v}})}, {(\mathfrak {B_{u}}\mathfrak {B_{v}})}, {(\mathfrak {B_{u}}\mathfrak {B_{v}})}, {(\mathfrak {B_{u}}+\mathfrak {B_{v}})^{2}}, \sqrt{\frac{\mathfrak {B_{u}}+\mathfrak {B_{v}}-2}{\mathfrak {B_{u}}\mathfrak {B_{v}}}}, \frac{1}{\sqrt{\mathfrak {B_{u}}+\mathfrak {B_{v}}}}$ and $\frac{2\sqrt{\mathfrak {B_{u}}\mathfrak {B_{v}}}}{\mathfrak {B_{u}}+\mathfrak {B_{v}}}$.

**Figure 1:**
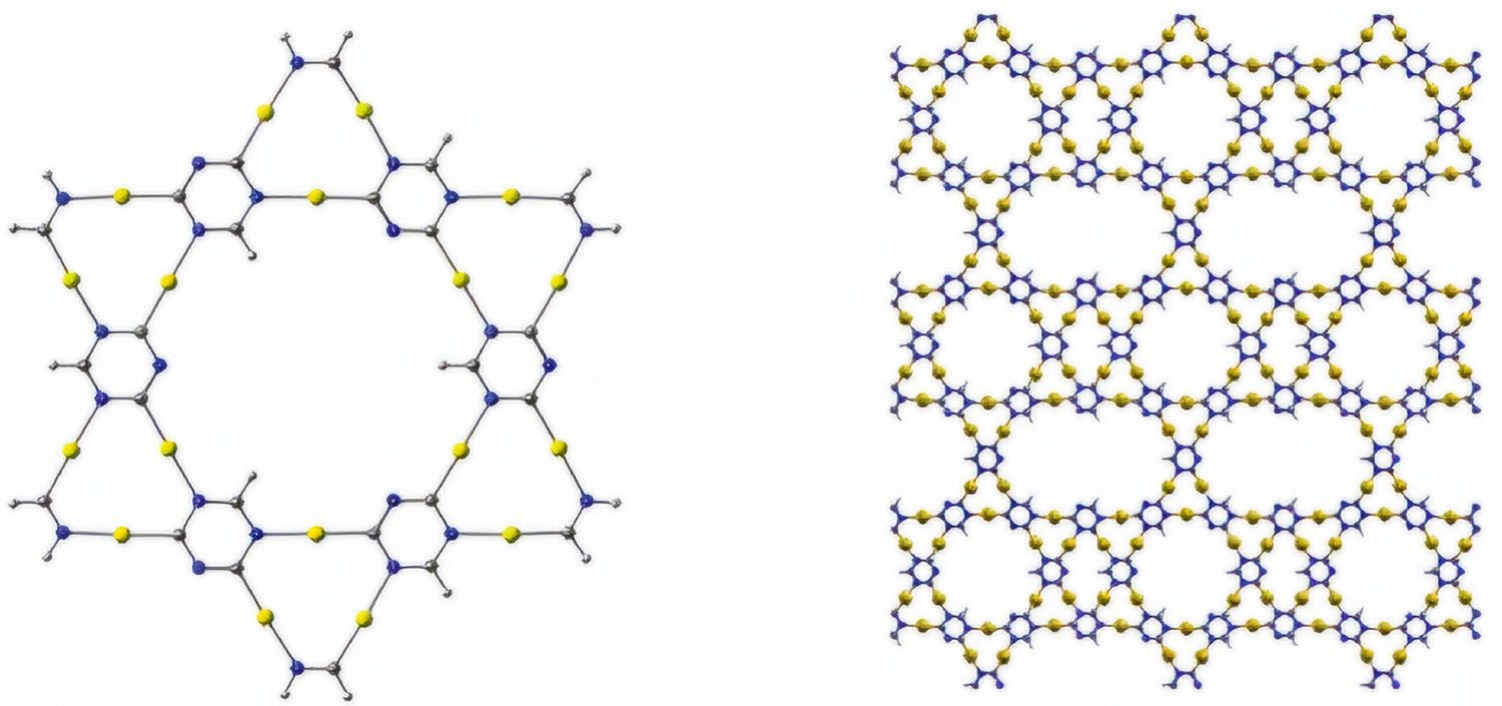
On the left side uni cell of a star gold structure and on the right side two-dimensional structure that is constructed based on the star-shaped molecule.

A beta-graphyne fragment and a two-dimensional star gold structure are produced by precisely positioning carbon atoms to form a unique structure with predefined bonding patterns. Beta-graphyne is a carbon allotrope with a honeycomb lattice structure similar to graphene, but with additional acetylenic linkages that give it a higher degree of sp-hybridization and unique electrical properties^25^. The unit cell of the two-dimensional star gold structure is shown on the left side of Fig. 1, and duplicates of the network are attached to the right side.

Because of its distinct structural and electrical characteristics, the two-dimensional star gold structure with a beta-graphyne fragment can be built and used in a variety of ways. In contrast to pristine graphene, the beta-graphyne fragment inserts localized pi-electron systems into the network, which can result in intriguing electrical behavior like increased conductivity or changed electronic band structure. Furthermore, the network’s star-shaped topology offers an attractive platform for the hosting of functional groups or nanoparticles at the ends of carbon chains, allowing for the customization of the network’s characteristics for particular uses like energy storage, catalysis, or sensing. Using Fig. 1 the edge set can be partitioned into 3 sets based on degree-based vertices.

$$\begin{aligned} E_{(1,3)}=\{\mathfrak {uv}\in E(SGN): \mathfrak {B_{u}}=1, \mathfrak {B_{v}}=3\} \\ E_{(2,3)}=\{\mathfrak {uv}\in E(SGN): \mathfrak {B_{u}}=2, \mathfrak {B_{v}}=3\} \\ E_{(3,3)}=\{\mathfrak {uv}\in E(SGN): \mathfrak {B_{u}}=3, \mathfrak {B_{v}}=3\} \end{aligned}$$

**Figure 2:**
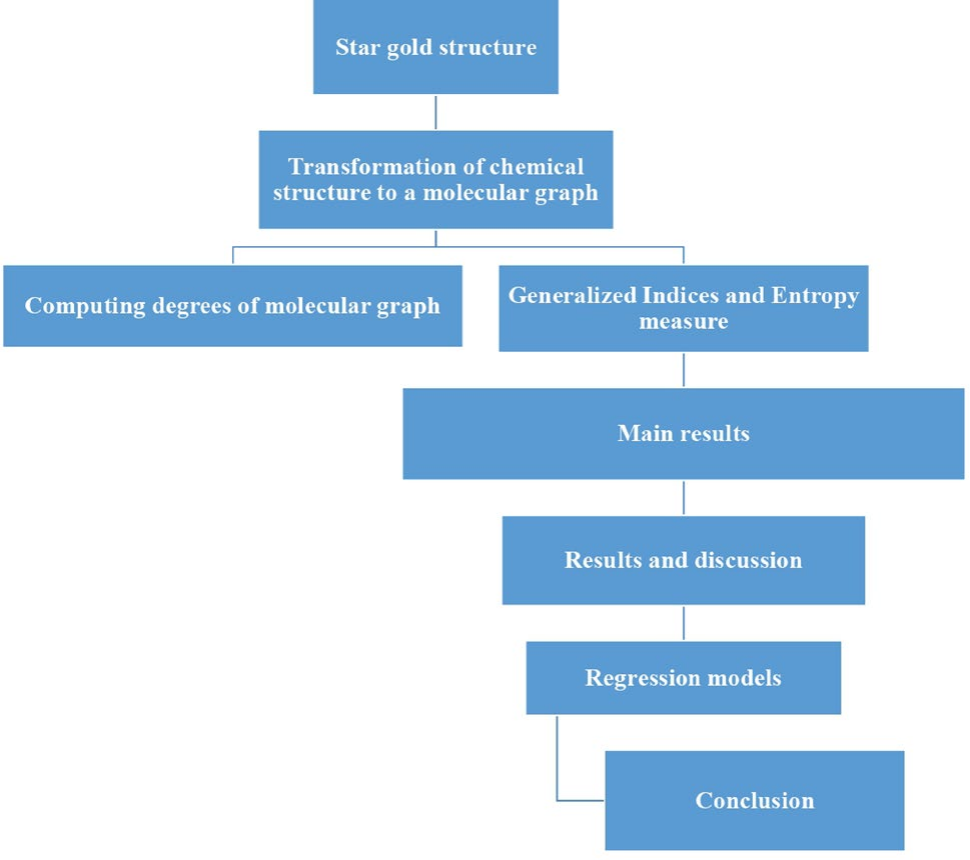
Flow chart of methodology.

The cardinality of $E_{(1,3)}$ is $6p+3q+9pq$, $E_{(2,3)}$ is $-4p-2q+54pq$ and $E_{(3,3)}$ is $-4p-2q+36pq$. The purpose of this study is to measure and examine the chosen structural characteristics of a two-dimensional star gold structure through the application of a variety of regression equations. The special objectives include evaluating the applicability of linear, logarithmic, and quadratic regression network equations and comparing their efficiencies. The methodology is shown in Fig. 2.

## Main results

### Theorem 1

*Let* $G\cong SGN$ *be a two-dimensional star gold structure. Then the Randic index of* *SGN is given as*:

$$\begin{aligned} \chi (SGN)= & {} =39.241pq + 0.497p + 0.248q. \end{aligned}$$

### Proof

We compute the Randic index using the above-computed edge partition based on degree and Eq. (1). The result is obtained as follows:

$$\begin{aligned} \chi (SGN)&=\sum _{\mathfrak {uv}\in E_{(1,3)}(SGN)}\frac{1}{\sqrt{\mathfrak {B_{u}}\mathfrak {B_{v}}}}+\sum _{\mathfrak {uv}\in E_{(2,3)}(SGN)}\frac{1}{\sqrt{\mathfrak {B_{u}}\mathfrak {B_{v}}}}+\sum _{\mathfrak {uv}\in E_{(3,3)}(SGN)}\frac{1}{\sqrt{\mathfrak {B_{u}}\mathfrak {B_{v}}}}\\ \chi (SGN)&=\frac{1}{\sqrt{1\times 3}}(6p+3q+9pq)+\frac{1}{\sqrt{2\times 3}}(-4p-2q+54pq)+\frac{1}{\sqrt{3\times 3}}(-4p-2q+36pq)\\ \chi (SGN)&=39.241pq + 0.497p + 0.248q. \end{aligned}$$

$$\square $$

### Theorem 2

*Let* $G\cong SGN$* be a two-dimensional star gold structure. Then the* $M_{1}(SGN)$* and* $M_{2}(SGN)$* of* *SGN are given as*:

$$\begin{aligned} M_{1}(SGN)= & {} 522pq - 20p - 10q\\ M_{2}(SGN)= & {} 675pq - 42p - 21q. \end{aligned}$$

### Proof

We compute the $M_{1}(SGN)$ and $M_{2}(SGN)$ indices using the above computed edge partition based on degree into Eqs. (2) and (3). The result is obtained as follows:

$$\begin{aligned} M_{1}(SGN)= & {} \sum _{\mathfrak {uv}\in E_{(1,3)}(SGN)}{(\mathfrak {B_{u}}+\mathfrak {B_{v}})}+\sum _{\mathfrak {uv}\in E_{(2,3)}(SGN)}{(\mathfrak {B_{u}}+\mathfrak {B_{v}})}+\sum _{\mathfrak {uv}\in E_{(3,3)}(SGN)}{(\mathfrak {B_{u}}+\mathfrak {B_{v}})}\\ M_{1}(SGN)= & {} (1+3)(6p+3q+9pq)+(2+3)(-4p-2q+54pq)+(3+3)(-4p-2q+36pq)\\ M_{1}(SGN)= & {} 522pq - 20p - 10q\\ M_{2}(SGN)= & {} \sum _{\mathfrak {uv}\in E_{(1,3)}(SGN)}{(\mathfrak {B_{u}}\mathfrak {B_{v}})}+\sum _{\mathfrak {uv}\in E_{(2,3)}(SGN)}{(\mathfrak {B_{u}}\mathfrak {B_{v}})}+\sum _{\mathfrak {uv}\in E_{(3,3)}(SGN)}{(\mathfrak {B_{u}}\mathfrak {B_{v}})}\\ M_{2}(SGN)= & {} (1\times 3)(6p+3q+9pq)+(2\times 3)(-4p-2q+54pq)+(3\times 3)(-4p-2q+36pq)\\ M_{2}(SGN)= & {} 675pq - 42p - 21q. \end{aligned}$$

$$\square $$

### Theorem 3

*Let* $G\cong SGN$* be a two-dimensional star gold structure. Then the* *HM and* (*ABC*)* indices of* *SGN are given as*:

$$\begin{aligned} HM(SGN)= & {} 2790pq - 148p - 74q\\ ABC(SGN)= & {} 69.53pq - 0.59p - 0.29q. \end{aligned}$$

### Proof

We compute the *HM* and (*ABC*) indices using the above computed edge partition based on degree into the Eqs. (4) and (5). The result is obtained as follows:

$$\begin{aligned} HM(SGN)= & {} \sum _{\mathfrak {uv}\in E_{(1,3)}(SGN)}{(\mathfrak {B_{u}}+\mathfrak {B_{v}})^{2}}+\sum _{\mathfrak {uv}\in E_{(2,3)}(SGN)}{(\mathfrak {B_{u}}+\mathfrak {B_{v}})^{2}}+\sum _{\mathfrak {uv}\in E_{(3,3)}(SGN)}{(\mathfrak {B_{u}}+\mathfrak {B_{v}})^{2}}\\ HM(SGN)= & {} (1+3)^{2}(6p+3q+9pq)+(2+3)^{2}(-4p-2q+54pq)+(3+3)^{2}(-4p-2q+36pq)\\ HM(SGN)= & {} 2790pq - 148p - 74q\\ ABC(SGN)= & {} \sum _{\mathfrak {uv}\in E_{(1,3)}(SGN)}{\sqrt{\frac{\mathfrak {B_{u}}+\mathfrak {B_{v}}-2}{\mathfrak {B_{u}}\mathfrak {B_{v}}}}}+\sum _{\mathfrak {uv}\in E_{(2,3)}(SGN)}{\sqrt{\frac{\mathfrak {B_{u}}+\mathfrak {B_{v}}-2}{\mathfrak {B_{u}}\mathfrak {B_{v}}}}}+\sum _{\mathfrak {uv}\in E_{(3,3)}(SGN)}{\sqrt{\frac{\mathfrak {B_{u}}+\mathfrak {B_{v}}-2}{\mathfrak {B_{u}}\mathfrak {B_{v}}}}}\\ ABC(SGN)= & {} \sqrt{\frac{{1+3-2}}{{1\times 3}}}(6p+3q+9pq)+\sqrt{{\frac{{2+3-2}}{{2\times 3}}}}(-4p-2q+54pq)+\sqrt{\frac{{3+3-2}}{{3\times 3}}}(-4p-2q+36pq)\\ ABC(SGN)= & {} 69.53pq - 0.59p - 0.29q. \end{aligned}$$

$$\square $$

### Theorem 4

*Let* $G\cong SGN$* be a two-dimensional star gold structure. Then the* *SCI*(*SGN*)* and* *GA*(*SGN*)* indices* (*SGN*)* are given as*:

$$\begin{aligned} SCI(SGN)= & {} 43.34pq - 0.42p - 0.21q\\ GA(SGN)= & {} 96.70pq - 2.72p - 1.36q. \end{aligned}$$

### Proof

We compute the *SCI*(*SGN*) and *GA*(*SGN*) indices using the above-computed edge partition based on degree into Eqs. (6) and (7). The result is obtained as follows:

$$\begin{aligned} SCI(SGN)= & {} \sum _{\mathfrak {uv}\in E_{(1,3)}(SGN)}\frac{1}{\sqrt{\mathfrak {B_{u}}+\mathfrak {B_{v}}}}+\sum _{\mathfrak {uv}\in E_{(2,3)}(SGN)}\frac{1}{\sqrt{\mathfrak {B_{u}}+\mathfrak {B_{v}}}}+\sum _{\mathfrak {uv}\in E_{(3,3)}(SGN)}\frac{1}{\sqrt{\mathfrak {B_{u}}+\mathfrak {B_{v}}}}\\ SCI(SGN)= & {} \frac{1}{\sqrt{1+3}}(6p+3q+9pq)+\frac{1}{\sqrt{2+3}}(-4p-2q+54pq)+\frac{1}{\sqrt{3+3}}(-4p-2q+36pq)\\ SCI(SGN)= & {} 43.34pq - 0.42p - 0.21q\\ GA(SGN)= & {} \sum _{\mathfrak {uv}\in E_{(1,3)}(SGN)}\frac{2\sqrt{\mathfrak {B_{u}}\mathfrak {B_{v}}}}{\mathfrak {B_{u}}+\mathfrak {B_{v}}}+\sum _{\mathfrak {uv}\in E_{(2,3)}(SGN)}\frac{2\sqrt{\mathfrak {B_{u}}\mathfrak {B_{v}}}}{\mathfrak {B_{u}}+\mathfrak {B_{v}}}+\sum _{\mathfrak {uv}\in E_{(3,3)}(SGN)}\frac{2\sqrt{\mathfrak {B_{u}}\mathfrak {B_{v}}}}{\mathfrak {B_{u}}+\mathfrak {B_{v}}}\\ GA(SGN)= & {} \frac{2\sqrt{1\times 3}}{1+3}(6p+3q+9pq)+\frac{2\sqrt{2\times 3}}{2+3}(-4p-2q+54pq)+\frac{2\sqrt{3\times 3}}{3+3}(-4p-2q+36pq)\\ GA(SGN)= & {} 96.70pq - 2.72p - 1.36q. \end{aligned}$$

$$\square $$

### Theorem 5

*Let* $G\cong SGN$* be a two-dimensional star gold structure. Then the Randic index entropy of* *SGN is given as*:

$$\begin{aligned} W_{\chi }((SGN))&=\log (39.241pq + 0.497p + 0.248q)-\left( \frac{-15.542pq + 0.445p + 0.222q}{39.241pq + 0.497p + 0.248q}\right) . \end{aligned}$$

### Proof

We compute the Randic index entropy using the above computed Randic index and edge partition based on degree into Eq. (9). The result is obtained as follows:

$$\begin{aligned} W_{\chi }(SGN)&=\log (\chi (SGN))-\frac{1}{(\chi (SGN))}\sum _{\mathfrak {uv}\in E(SGN)}\mathfrak {f(uv)}\log {\mathfrak {f(\mathfrak {uv})}}\\ W_{\chi }(SGN)&=\log (39.241pq + 0.497p + 0.248q)-\left( \frac{1}{39.241pq + 0.497p + 0.248q}\right) \left( \frac{1}{\sqrt{3}}(6p+3q+9pq)\log {(\frac{1}{\sqrt{3}})}\right. \\ \end{aligned}$$

$$\begin{aligned}{}&\left. +\frac{1}{\sqrt{6}}(-4p-2q+54pq)\log {(\frac{1}{\sqrt{6}})}+\frac{1}{\sqrt{9}}(-4p-2q+36pq)\log {(\frac{1}{\sqrt{9}})}\right) \\ W_{\chi }(SGN)&=\log (39.241pq + 0.497p + 0.248q)-\left( \frac{1}{39.241pq + 0.497p + 0.248q}\right) \left( (1.73)(6p+3q+9pq)(0.23)\right. \\&\left. +(0.4)(-4p-2q+54pq)(-0.36)+(0.33)(-4p-2q+36pq)(-0.48)\right) \\ W_{\chi }(SGN)&=\log (39.241pq + 0.497p + 0.248q)-\left( \frac{-15.542pq + 0.445p + 0.222q}{39.241pq + 0.497p + 0.248q}\right) . \end{aligned}$$

$$\square $$

### Theorem 6

*Let* $G\cong SGN$* be a two-dimensional star gold structure. Then the* $W_{M_{1}}(SGN)$* and* $W_{M_{2}}(SGN)$* index entropy of* *SGN are given as*:

$$\begin{aligned} W_{M_{1}}(SGN)&=\log (522pq - 20p - 10q)-\left( \frac{378.47pq - 18.20559030p - 9.102q}{522pq - 20p - 10q}\right) \\ W_{M_{2}}(SGN)&=\log (675pq - 42p - 21q)-\left( \frac{574.17pq - 44.44p - 22.220q}{675pq - 42p - 21q}\right) . \end{aligned}$$

### Proof

We compute the $W_{M_{1}}(SGN)$ and $W_{M_{2}}(SGN)$ index entropy using the above computed $W_{M_{1}}(SGN)$ and $W_{M_{2}}(SGN)$ indices and edge partition based on degree into Eq. (9). The result is obtained as follows:

$$\begin{aligned} W_{M_{1}}(SGN)&=\log (M_{1}(SGN))-\frac{1}{(M_{1}(SGN))}\sum _{\mathfrak {uv}\in E(SGN)}\mathfrak {f(uv)}\log {\mathfrak {f(\mathfrak {uv})}}\\ W_{M_{1}}(SGN)&=\log (522pq - 20p - 10q)-\left( \frac{1}{522pq - 20p - 10q}\right) \left( (4)(6p+3q+9pq)\log (4)\right. \\&\left. +(5)(-4p-2q+54pq)\log (5)+(6)(-4p-2q+36pq)\log (6)\right) \\ W_{M_{1}}(SGN)&=\log (522pq - 20p - 10q)-\left( \frac{378.47pq - 18.20559030p - 9.102q}{522pq - 20p - 10q}\right) \\ W_{M_{2}}(SGN)&=\log (M_{2}(SGN))-\frac{1}{(M_{2}(SGN))}\sum _{\mathfrak {uv}\in E(SGN)}\mathfrak {f(uv)}\log {\mathfrak {f(\mathfrak {uv})}}\\ W_{M_{2}}(SGN)&=\log (675pq - 42p - 21q)-\left( \frac{1}{675pq - 42p - 21q}\right) \left( (3)(6p+3q+9pq)\log (3)\right. \\&\left. +(6)(-4p-2q+54pq)\log (6)+(9)(-4p-2q+36pq)\log (9)\right) \\ W_{M_{2}}(SGN)&=\log (675pq - 42p - 21q)-\left( \frac{574.17pq - 44.44p - 22.220q}{675pq - 42p - 21q}\right) . \end{aligned}$$

$$\square $$

### Theorem 7

*Let* $G\cong SGN$* be a two-dimensional star gold structure. Then the* $W_{HM}(SGN)$* and* $W_{ABC}(SGN)$* indices entropy of* *SGN are given as*:

$$\begin{aligned} W_{HM}(SGN)&=\log (2790pq - 148p - 74q)-\frac{4077.58pq - 248.30p - 124.15q}{2790pq - 148p - 74q}\\ W_{ABC}(SGN)&=\log (69.53pq - 0.59p - 0.29q)-\frac{-10.62pq + 0.46p + 0.23q}{69.53pq - 0.59p - 0.29q}. \end{aligned}$$

### Proof

We compute the $W_{HM}(SGN)$ and $W_{ABC}(SGN)$ entropy using the above computed *HM*(*SGN*) and *ABC*(*SGN*) and edge partition based on degree into Eq. (9). The result is obtained as follows:

$$\begin{aligned} W_{HM}(SGN)&=\log (HM(SGN))-\frac{1}{(HM(SGN))}\sum _{\mathfrak {uv}\in E(SGN)}\mathfrak {f(uv)}\log {\mathfrak {f(\mathfrak {uv})}}\\ W_{HM}(SGN)&=\log (2790pq - 148p - 74q)-\left( \frac{1}{2790pq - 148p - 74q}\right) \\&\times \left( (16)(6p+3q+9pq)\log (16)+(25)(-4p-2q+54pq)\log (25)+(36)(-4p-2q+36pq)\log (36)\right) \\ W_{HM}(SGN)&=\log (2790pq - 148p - 74q)-\frac{4077.58pq - 248.30p - 124.15q}{2790pq - 148p - 74q}\\ W_{ABC}(SGN)&=\log (ABC(SGN))-\frac{1}{(ABC(SGN))}\sum _{\mathfrak {uv}\in E(SGN)}\mathfrak {f(uv)}\log {\mathfrak {f(\mathfrak {uv})}}\\ \end{aligned}$$

$$\begin{aligned} W_{ABC}(SGN)&=\log (69.53pq - 0.59p - 0.29q)\\&-\left( \frac{1}{69.53pq - 0.59p - 0.29q}\right) \left( (\frac{2}{3})(6p+3q+9pq)\log (\frac{2}{3})+(\frac{3}{6})(-4p-2q+54pq)\log (\frac{3}{6})\right. \\&\left. +(\frac{4}{9})(-4p-2q+36pq)\log (\frac{4}{9})\right) \\ W_{ABC}(SGN)&=\log (69.53pq - 0.59p - 0.29q)-\frac{-10.62pq + 0.46p + 0.23q}{69.53pq - 0.59p - 0.29q}. \end{aligned}$$

$$\square $$

### Theorem 8

*Let* $G\cong SGN$* be a two-dimensional star gold structure. Then the* $W_{SCI}(SGN)$* and* $W_{GA}(SGN)$* entropy of* *SGN are given as*:

$$\begin{aligned} W_{SCI}(SGN)&=\log (43.34pq - 0.42p - 0.21q)-\frac{-15pq + 0.35p + 0.17q}{43.34pq - 0.42p - 0.21q}\\ W_{GA}(GSGN)&=\log (96.70pq - 2.72p - 1.36q)-\frac{-0.95pq - 0.28p - 0.14q}{96.70pq - 2.72p - 1.36q}. \end{aligned}$$

### Proof

We compute the $W_{SCI}(SGN)$ and $W_{GA}(SGN)$ entropy using the above-computed sum-connectivity index and geometric arithmetic index and edge partition based on degree into Eq. (9). The result is obtained as follows:

$$\begin{aligned} W_{SCI}(SGN)&=\log (SCI(SGN))-\frac{1}{(SCI(SGN))}\sum _{\mathfrak {uv}\in E(SGN)}\mathfrak {f(uv)}\log {\mathfrak {f(\mathfrak {uv})}}\\ W_{SCI}(SGN)&=\log (43.34pq - 0.42p - 0.21q)\\&-\left( \frac{1}{43.34pq - 0.42p - 0.21q}\right) \left( (\frac{1}{\sqrt{4}})(6p+3q+9pq)\log (\frac{1}{\sqrt{4}})\right. \\&\left. +(\frac{1}{\sqrt{5}})(-4p-2q+54pq)\log (\frac{1}{\sqrt{5}})+(\frac{1}{\sqrt{6}})(-4p-2q+36pq)\log (\frac{1}{\sqrt{6}})\right) \\ W_{SCI}(SGN)&=\log (43.34pq - 0.42p - 0.21q)-\frac{-15pq + 0.35p + 0.17q}{43.34pq - 0.42p - 0.21q}\\ W_{GA}(SGN)&=\log (GA(SGN))-\frac{1}{(GA(SGN))}\sum _{\mathfrak {uv}\in E(SGN)}\mathfrak {f(uv)}\log {\mathfrak {f(\mathfrak {uv})}}\\ W_{GA}(SGN)&=\log (96.70pq - 2.72p - 1.36q)\\&-\left( \frac{1}{96.70pq - 2.72p - 1.36q}\right) \left( (\frac{2\sqrt{3}}{4})(6p+3q+9pq)\log (\frac{2\sqrt{3}}{4})\right. \\&\left. +(\frac{2\sqrt{6}}{5})(-4p-2q+54pq)\log (\frac{2\sqrt{6}}{5})+(\frac{2\sqrt{9}}{6})(-4p-2q+36pq)\log (\frac{2\sqrt{9}}{6})\right) \\ W_{GA}(SGN)&=\log (96.70pq - 2.72p - 1.36q)-\frac{-0.95pq - 0.28p - 0.14q}{96.70pq - 2.72p - 1.36q}. \end{aligned}$$

$$\square $$

## Results and discussion

**Figure 3:**
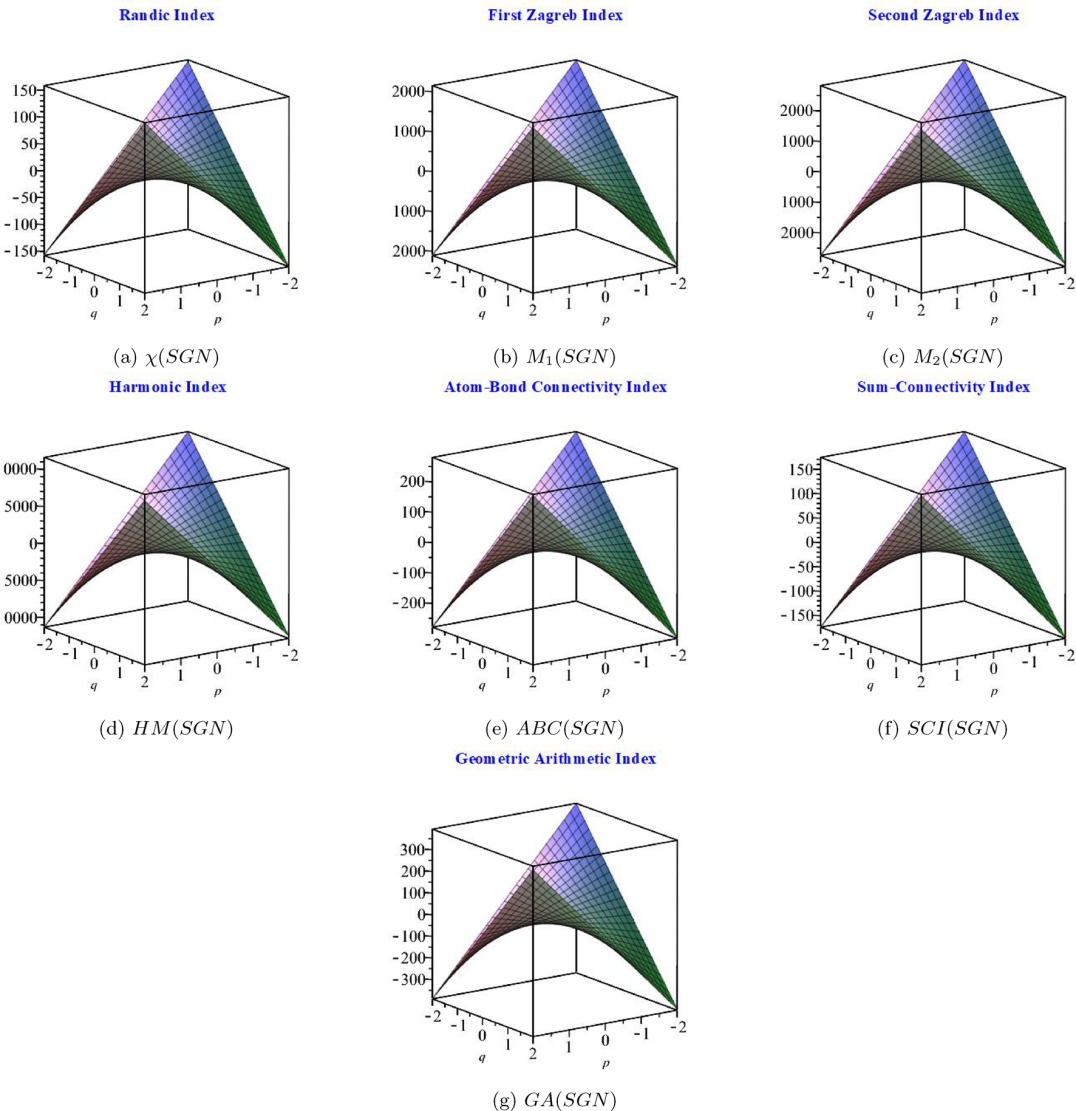
Graphical behaviour of (**a**) $\chi (SGN)$, (**b**) $M_{1}(SGN)$, (**c**) $M_{2}(SGN)$, (**d**) *HM*(*SGN*), (**e**) *ABC*(*SGN*), (**f**) *SCI*(*SGN*) and (**g**) *GA*(*SGN*).

In this section, we compute the numerical values of graph entropies and topological indices of the two-dimensional star gold structure for some of the values of *p* and *q*. We also generate 3D graphs to compare the outcomes that we have obtained. Here, the base-10 logarithm is used in the computations. The numerical values of the topological indices are shown in Table 1. The order of the topological index values is as follows: $HM(SGN)>M_{2}(SGN)>M_{1}(SGN)>GA(SGN)>ABC(SGN)>SCI(SGN)>\chi (SGN)$. In the graphical comparison, we plot 3D indices graphs, as shown in Fig. 3. We observe that when we increase and decrease the values of *p* and *q*, the values of each index increase and decrease, respectively.

**Table 1:** Numerical comparison between topological indices.

$Indices\backslash [p,q]$	$[-2,-2]$	$[-1,-1]$	[1, 1]	[2, 2]
$\chi (SGN)$	155.47	38.49	39.98	158.45
$M_{1}(SGN)$	2148	552	492	2028
$M_{2}(SGN)$	2826	738	612	2574
*HM*(*SGN*)	11604	3012	2568	10716
*ABC*(*SGN*)	279.91	70.42	68.63	276.34
*SCI*(*SGN*)	174.65	43.97	42.71	172.12
*GA*(*SGN*)	394.98	100.78	92.61	378.64

**Figure 4:**
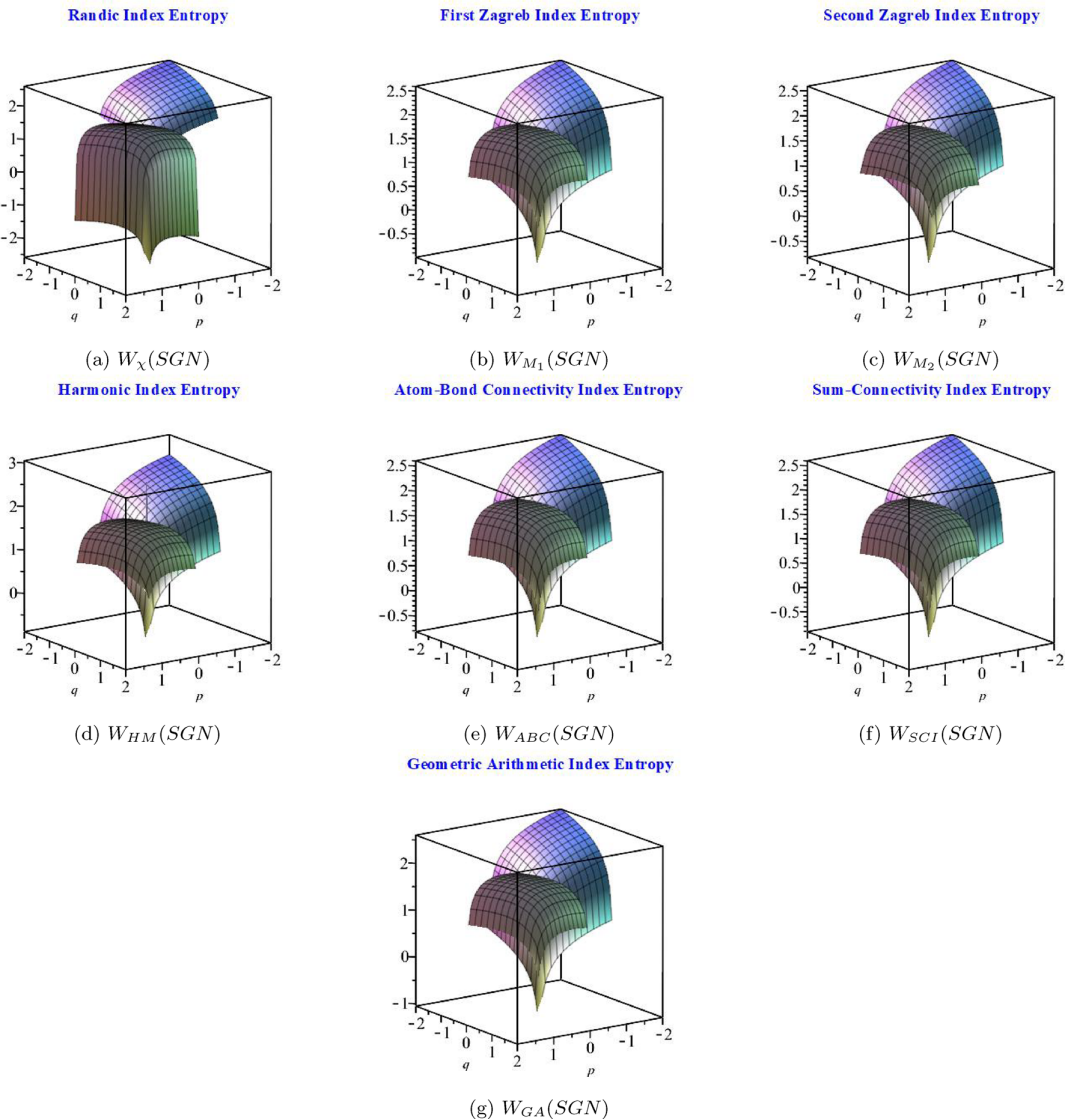
Graphical behaviour of (**a**) $W_{\chi }(SGN)$, (**b**) $W_{M_{1}}(SGN)$, (**c**) $W_{M_{2}}(SGN)$, (**d**) $W_{HM}(SGN)$, (**e**) $W_{ABC}(SGN)$, (**f**) $W_{SCI}(SGN)$ and (**g**) $W_{GA}(SGN)$.

The calculated numerical values of entropies are shown in Table 2, where a clear trend can be seen: the numerical values of entropy drop as the values of *p* and *q* rise. This finding highlights the connection between the entropy that results from a system’s probability distribution and that relationship. Figure 4 illustrate the graphical behaviour of several entropies, which helps to clarify this relationship even further. These graphic depictions help to clarify the complex dynamics of entropy under different conditions, which contributes to a better comprehension of the fundamental ideas behind information theory.

**Table 2:** Numerical comparison between entropies.

$Indices\backslash [p,q]$	$[-2,-2]$	$[-1,-1]$	[1, 1]	[2, 2]
$W_{\chi }(SGN)$	2.600099055	2.006472339	1.973913164	2.583825355
$W_{M_{1}}(SGN)$	2.601822368	2.006834748	1.978220368	2.587509362
$W_{M_{2}}(SGN)$	2.591299546	1.999723029	1.957486716	2.570142342
$W_{HM}(SGN)$	2.594835275	2.001418251	1.966790788	2.577494147
$W_{ABC}(SGN)$	2.603730376	2.008355348	1.981206816	2.590157302
$W_{SCI}(SGN)$	2.603583841	2.008153599	1.981187194	2.590102108
$W_{GA}(SGN)$	2.604032470	2.008545193	1.981719837	2.590621383

## Regression models

Regression models are used by statisticians to depict the relationship between a dependent variable and one or more independent variables. The three most commonly used forms of regression models are logarithmic, quadratic, and linear. In linear regression, a linear relationship between the dependent variable *Q* and one or more independent variables *S* is assumed.

$$\begin{aligned} Q&=\beta _{0}+\beta _{1}S_{1}+\cdot +\beta _{n}S_{n}+\varepsilon \end{aligned}$$

Where $\varepsilon$ represents error terms. In our case, it is zero, and $\beta _{0}, \beta _{1}, \cdot , \beta _{n}$ represent regression coefficients. By minimising the sum of squared residuals, linear regression seeks to identify the coefficients that most closely match the observed data points. By permitting a curved relationship between the dependent and independent variables, quadratic regression expands on the capabilities of linear regression. It entails fitting the data into a quadratic equation. The following is the model equation:

$$\begin{aligned} Q&=\beta _{0}+\beta _{1}S+\beta _{2}S^{2}+\varepsilon \end{aligned}$$

The following is the logarithmic regression model equation:

$$\begin{aligned} Q&=\beta _{0}+\beta _{1}\ln (S)+\varepsilon \end{aligned}$$

To evaluate the effectiveness and importance of the model, regression analysis depends on several key metrics. Greater values imply a more suitable match. R-squared is a statistic that shows how much of the variance of the dependent variable can be explained by the independent variables. Its values vary from 0 to 1. Through a comparison between the residual variability and the variability explained by the regression model, the F-value assesses the overall significance of the model and helps determine whether it is statistically significant overall.

**Figure 5:**
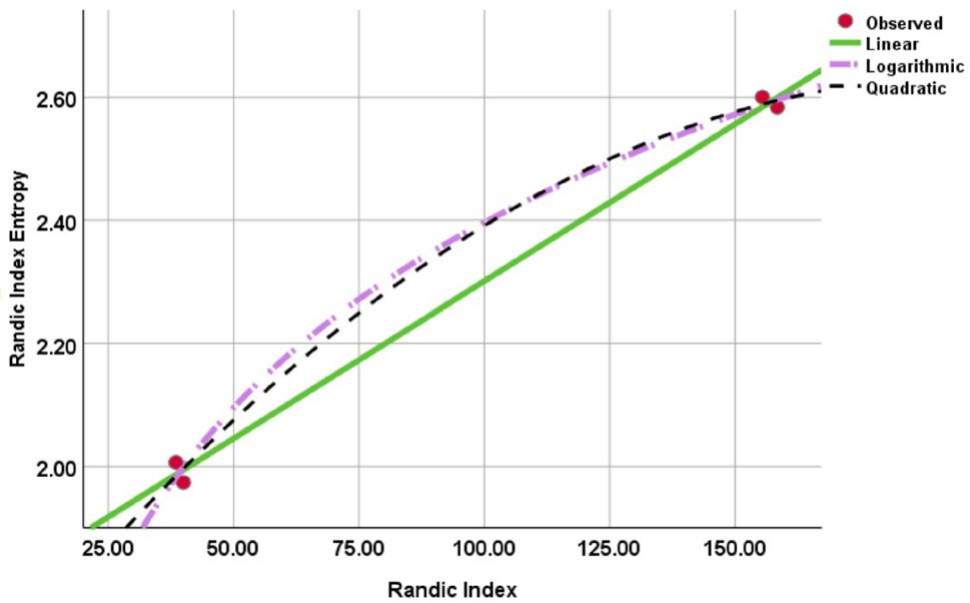
Curve fitting between Randic Index and Randic Index Entropy.

It has been discovered that the quadratic and logarithmic regression models both maximize the R square value when constructing a regression model between $W_{\chi }(SGN)$ and the Randic index. Furthermore, the F-value maximizes in the logarithmic regression model. However, the significant value of the logarithmic regression model is somewhat lower than that of the other models. This suggests that there may be a slight compromise in the relevance of the logarithmic regression model, despite the fact that it displays the best correlation between the Randic entropy and the Randic index. This result is illustrated in Table 3 and Fig. 5, which demonstrate that of the models studied, the logarithmic regression model has the best overall fit and explanatory power.

**Table 3:** Regression models and statistical parameters for $\chi (SGN)$.

Models	Equations	$R^{2}$	*F*	*Sig*.
Linear	$W_{\chi }(SGN)$=$0.005[\chi (SGN)]+1.790$	0.996	554.878	0.02
Quadratic	$W_{\chi }(SGN)$=$2.60[\chi (SGN)]^{2}+0.010[\chi (SGN)]+1.630$	0.998	144.507	0.059
Logarithmic	$W_{\chi }(SGN)$=$0.433\ln [\chi (SGN)]+0.400$	0.998	480.957	0.002

**Figure 6:**
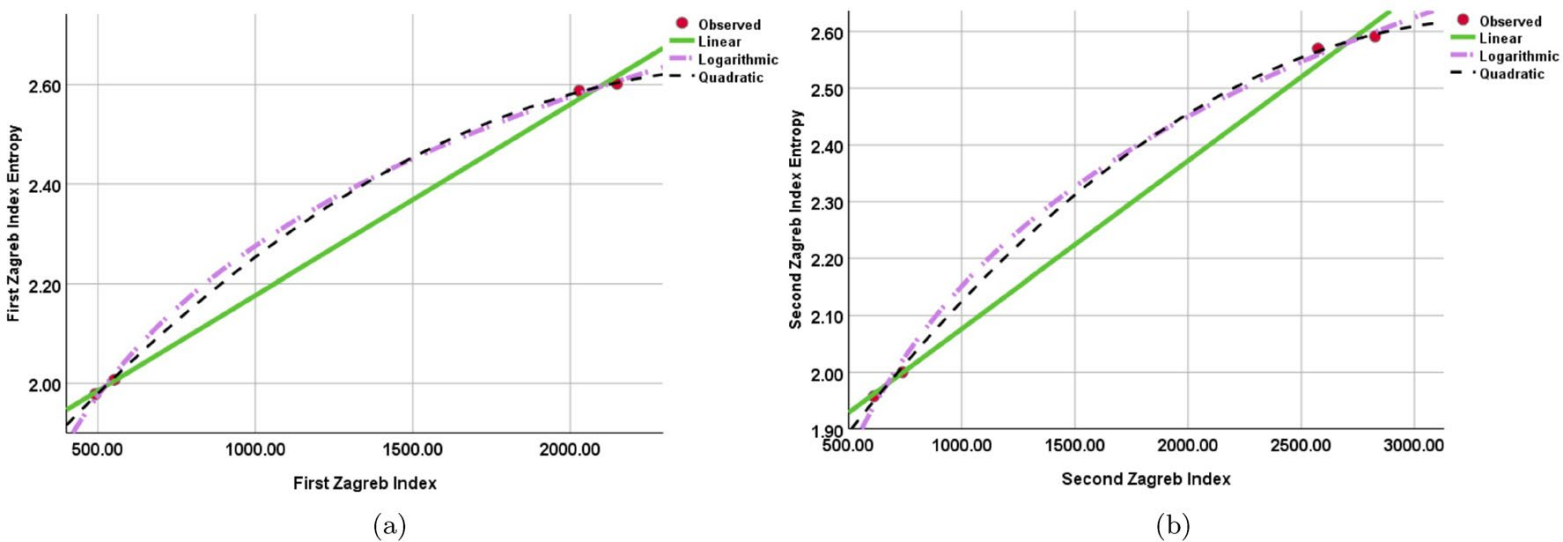
Curve fitting between (**a**) First Zagreb Index vs First Zagreb Index Entropy. (**b**) Second Zagreb Index vs Second Zagreb Index Entropy.

It is found that while building a regression model between the $W_{M_{1}}(SGN)$ and ${M_{1}}(SGN)$, and the $W_{M_{2}}(SGN)$ and ${M_{2}}(SGN)$ indices, the quadratic regression models maximize the R square value. Moreover, under the quadratic regression models, the F-value maximizes. Nevertheless, when compared to the other models, the logarithmic regression model’s significance value is comparatively smaller. This implies that although the logarithmic regression model shows the best correlation between the $W_{M_{1}}(SGN)$ and $W_{M_{1}}(SGN)$ and the ${M_{1}}(SGN)$ and ${M_{1}}(SGN)$ indices, there may be a small compromise in its relevance. Tables 4 and 5 and Fig. 6 highlight this finding, showing that the quadratic regression models have the best overall fit and explanatory power among the models examined.

**Table 4:** Regression models and statistical parameters for for $M_{1}(SGN)$.

Models	Equations	$R^{2}$	*F*	*Sig*.
Linear	$W_{M_{1}}(SGN)$=$0.001[M_{1}(SGN)]+1.793$	0.999	1392.756	0.001
Quadratic	$W_{M_{1}}(SGN)$=$-1.473[M_{1}(SGN)]^{2}+0.001[M_{1}(SGN)]+1.632$	1	4205.111	0.011
Logarithmic	$W_{M_{1}}(SGN)$=$0.433\ln [M_{1}(SGN)]-717$	0.999	2567.314	0.0001

**Table 5:** Regression models and statistical parameters for $M_{2}(SGN)$.

Models	Equations	$R^{2}$	*F*	*Sig*.
Linear	$W_{M_{2}}(SGN)$=$0.0002[M_{2}(SGN)] + 1.781$	0.996	500.634	0.002
Quadratic	$W_{M_{2}}(SGN)$=$1.618 + 0.00059[M_{2}(SGN)] -8.796[M_{2}(SGN)]^{2}$	0.999	923.781	0.023
Logarithmic	$W_{M_{2}}(SGN)$=$0.431\ln [M_{2}(SGN)]-0.826$	0.997	778.435	0.001

**Figure 7:**
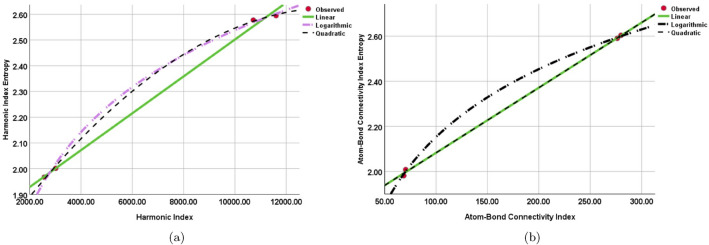
Curve fitting between (**a**) Harmonic Index vs Harmonic Index Entropy. (**b**) Atom-Bond Index vs Atom-Bond Index Entropy.

It is found that while building a regression model between the $W_{HM}(SGN)$ and $W_{ABC}(SGN)$ entropy and the *HM*(*SGN*) and *ABC*(*SGN*) indices, the quadratic regression models maximize the R square value. Moreover, under the quadratic regression models, the F-value maximizes. Nevertheless, when compared to the other models, the logarithmic regression model’s significance value is comparatively smaller. This implies that although the logarithmic regression model shows the best correlation between the $W_{HM}(SGN)$ and $W_{ABC}(SGN)$ entropy and the *HM*(*SGN*) and *ABC*(*SGN*) indices, there may be a small compromise in its relevance. Tables 6 and 7 and Fig. 7 highlight this finding, showing that the quadratic regression models have the best overall fit and explanatory power among the models examined.

**Table 6:** Regression models and statistical parameters for *HM*(*SGN*).

Models	Equations	$R^{2}$	*F*	*Sig*.
Linear	$W_{HM}(SGN)$=$7.165[HM(SGN)]+1.785$	0.999	670.274	0.001
Quadratic	$W_{HM}(SGN)$=$-5.90[HM(SGN)]^{2}+0.001[HM(SGN)]+1.624$	1	1068.92	0.022
Logarithmic	$W_{HM}(SGN)$=$0.432\ln [HM(SGN)]-1.439$	0.998	983.640	0.001

**Table 7:** Regression models and statistical parameters for *ABC*(*SGN*).

Models	Equations	$R^{2}$	*F*	*Sig*.
Linear	$W_{ABC}(SGN)$=$0.003[ABC(SGN)]+1.794$	0.999	2938.788	0.001
Quadratic	$W_{ABC}(SGN)$=$-1.80[ABC(SGN)]^{2}+0.003[ABC(SGN)]+1.794$	1	2938.4	0.001
Logarithmic	$W_{ABC}(SGN)$=$0.434\ln [ABC(SGN)]+1.52$	0.999	4553.232	0.001

**Figure 8:**
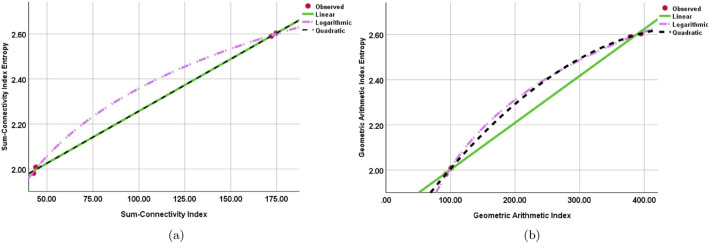
Curve fitting between (**a**) Sum-Connectivity Index vs Sum-Connectivity Index Entropy. (**b**) Geometric–Arithmetic Index vs Geometric–Arithmetic Index Entropy.

It is found that while building a regression model between the $W_{SCI}(SGN)$ and $W_{GA}(SGN)$ and the *SCI*(*SGN*) and *GA*(*SGN*) indices, the quadratic regression models maximize the R square value. Moreover, under the quadratic regression models, the F-value maximizes. Nevertheless, when compared to the other models, the logarithmic regression model’s significance value is comparatively smaller. This implies that although the logarithmic regression model shows the best correlation between the $W_{SCI}(SGN)$ and $W_{GA}(SGN)$ and the *SCI*(*SGN*) and *GA*(*SGN*) indices, there may be a small compromise in its relevance. Tables 8 and 9 and Fig. 8 highlight this finding, showing that the quadratic regression models have the best overall fit and explanatory power among the models examined.

**Table 8:** Regression models and statistical parameters for *SCI*(*SGN*).

Models	Equations	$R^{2}$	*F*	*Sig*.
Linear	$W_{SCI}(SGN)$=$0.004[SCI(SGN)]-1.794$	0.998	1920.834	0.001
Quadratic	$W_{SCI}(SGN)$=$-2.80[SCI(SGN)]^{2}+0.002[SCI(SGN)]-1.794$	1	2938.4	0.001
Logarithmic	$W_{SCI}(SGN)$=$0.433\ln [SCI(SGN)]+1.53$	0.999	5432.232	0.002

**Table 9:** Regression models and statistical parameters for *GA*(*SGN*).

Models	Equations	$R^{2}$	*F*	*Sig*.
Linear	$W_{GA}(SGN)$=$0.002[GA(SGN)]+2.794$	0.998	1310.345	0.002
Quadratic	$W_{GA}(SGN)$=$-1.30[GA(SGN)]^{2}+0.001[GA(SGN)]+2.794$	1	3048.4	0.001
Logarithmic	$W_{GA}(SGN)$=$0.431\ln [GA(SGN)]+1.33$	0.999	4432.232	0.001

## Conclusion

We computed several indices and then entropy in order to examine the structural characteristics of the star gold structure. In order to look into the relationship between entropy and indices, we also constructed regression models. Our findings indicate that the entropy and produced indices of the Star Gold graph fit well into the logarithmic and quadratic regression models. Specifically, a strong connection was seen between the two models, suggesting that the concept of entropy effectively describes the intricacy and interconnectivity of the network as expressed by the calculated indices. Our results expand on the structural aspects of the star gold structure and show that regression modeling is a valuable tool for locating critical links within complex network systems. Further research could look at other factors influencing network dynamics and broaden the scope of the study to include other kinds of networks in order to obtain a deeper knowledge of the traits and behaviours of other network types.

## Future directions

Consequently, several directions for further study become apparent:

- Diverse Network Types: Examine how entropy measure can be applied to a wider variety of network types, such as scale-free networks, small-world networks, and real-world networks from other domains.
- Nonlinear Analysis Techniques: Beyond the constraints of linear regression, investigate nonlinear analysis techniques to capture more intricate correlations between entropy metrics and network properties.

## Data Availability

The datasets used and/or analyzed during the current study available from the corresponding author on reasonable request.
